# Highly Reliable Fuzzy-Logic-Assisted AODV Routing Algorithm for Mobile Ad Hoc Networks

**DOI:** 10.3390/s21175965

**Published:** 2021-09-06

**Authors:** Jiamin Li, Mengling Wang, Pengcheng Zhu, Dongming Wang, Xiaohu You

**Affiliations:** National Mobile Communications Research Laboratory, Southeast University, Nanjing 210096, China; lijiamin@seu.edu.cn (J.L.); wangml@seu.edu.cn (M.W.); wangdm@seu.edu.cn (D.W.); xhyu@seu.edu.cn (X.Y.)

**Keywords:** MANET, fuzzy logic, AODV, FL-AODV, reliability, routing protocol

## Abstract

Due to the noncentered, self-organizing, and self-healing characteristics, mobile ad hoc networks (MANET) have been more and more widely used as an alternative access technology for regions having no fixed infrastructure. On-demand routing protocols (e.g., ad hoc on-demand distance vector (AODV)) are used to cope with the rapidly changing topology of MANET and reduce the network overhead. Taking delay, stability, and remaining energy of nodes into consideration, a fuzzy-logic-assisted AODV (FL-AODV) routing algorithm is proposed in this paper to further improve the reliability of the route in MANET. In the route discovery phase, the node with the highest reliability is selected as the relay node, and the route with the highest accumulated reliability is reserved for data transmission. Simulation results show that, compared with the traditional AODV protocol and the fuzzy logic routing algorithm (FLRA), the proposed routing protocol has higher reliability without increasing delay, i.e., better link connectivity and longer route life. The average routing reliability is about 18% higher than AODV while the average delay is the same low when the number of node greater than 70.

## 1. Introduction

Mobile ad hoc networks (MANET) consisting of a group of mobile nodes is an infrastructureless self-organizing and self-healing multihop network [1,2]. It can be applied to environments such as vehicles and drones, and forms vehicular ad hoc networks (VANET) [3] and flying ad hoc networks (FANET) [4], respectively.

In view of the mobility of nodes and the dynamically changing topological structure, it is important to design routing protocols with high reliability and low latency in MANET. According to when and how the routes are discovered, routing protocols can be divided into table-driven and on-demand routing [5]. Table-driven routing protocol is suitable for the networks with slowly changing topologies, and each node in the network must maintain routing tables to the other nodes. The advantage is lower delay and packet loss rate, while the disadvantage is higher control overhead and thus it is only suitable for small-scale networks. The traditional table-driven routing protocols include Optimized Link State Routing (OLSR) and Destination Sequenced Distance Vector (DSDV) [6]. For networks with rapidly changing topologies, on-demand routing protocol is a better choice [7]. Only when data transmission is needed, routing discovery process and the establishment of routing table information are carried out. The advantage is lower control overhead, better adaptability to rapidly changing topologies and lower performance requirements for mobile nodes. It comes at the cost of higher routing delay. Traditional on-demand routing protocols include ad hoc on-demand distance vector (AODV) and dynamic source routing (DSR) [6].

Many improved on-demand routing protocols have been proposed. The authors of [8] proposed energy and quality of service (QoS) supported AODV (EQ-AODV), which enhances network life and reduces network load and end-to-end delay. Ref. [9] proposed a multipath routing algorithm based on distance and energy consumption constraints in wireless sensor networks, which selects the neighbor node with the largest weight to transmit data. However, in a dynamic topology environment, it is complicated to accurately calculate the probability of being selected as a relay node determined by multiple performance metrics (e.g., delay and stability). Therefore, in this paper, we propose fuzzy logic to comprehensively deal with the factors that are not precise in delay, stability, and residual energy and screen out high-reliability and low-delay routing paths. Fuzzy logic is a well-known artificial intelligence tool that can combine human experience and digital information to solve the decision making process in a dynamic and constantly changing system. In [10], fuzzy logic was used to infer the weights of different objective functions according to the different type of service required by users to solve a multiobjective resource optimization problem in VANETs. The authors of [11] proposed to extend the life of the network based on fuzzy logic by considering energy efficiency and energy consumption balancing of sensor nodes simultaneously.

Based on fuzzy logic, many improved routes have also been proposed. Considering different contradictory indicators, a fuzzy logic framework was proposed in [12] to select the node with a output value of fuzzy logic system greater than the threshold in route request packet (RREQ) as relay node during the route discovery process. Similarly, in [13,14], the most suitable next-hop node was selected among the candidate forwarders for packet forwarding based on geographic routing. The authors of [15] proposed to use fuzzy logic only to compromise with flight autonomy, mobility level, and received signal strength indicator of nodes to establish communication routing with a longer active period in FANET. In order to reduce the number of hops caused by the optimal fuzzy logic output value node as the relay node, the authors of [16] proposed to combine the reinforcement learning and fuzzy logic into FANET to achieve fewer hops by considering the suboptimal nodes. The authors of [17] made use of fuzzy logic to determine the optimal route, and took into consideration multiple parameters to select routing relay nodes, but fuzzy logic was only used in the destination node. Improving the energy efficiency of routing algorithms through fuzzy logic controller was proposed in [18]. However, these works mainly focused on using fuzzy logic to determine the optimal node without considering reliability and hops simultaneously, which increases the probability of link interruption and delay.

In order to determine a route of high reliability and low latency in the topology of dynamic rapid changes, we propose to use fuzzy logic based on the AODV of the shortest path to consider multiple parameters to enhance routing reliability and delay concurrently. In addition, considering route constructed by more stable and more surplus energy nodes can maintain longer activity period to achieve reliable transmission of data packets. Therefore, delay, stability, and residual energy are selected as the evaluation of node reliability.

In this paper, we propose a highly reliable fuzzy-logic-assisted AODV routing (FL-AODV) algorithm. The main contributions of this paper are summarized as follows:

- Reliability of route is described by delay, stability, and remaining energy of nodes. Fuzzy logic is used to fuzzy the considered performance metrics and thus to realize the uncertainty conversion between fuzzy language concepts and quantitative values.
- Based on node reliability, the fuzzy-logic-assisted AODV routing algorithm is proposed. The node with the largest reliability is added to the routing table during route discovery process with AODV to determine a route with high reliability and low latency. Simulation experiments have been implemented to verify the effectiveness of the proposed algorithm.

The rest of this paper is organized as follows. In Section , we introduce the system model of this paper and review the traditional AODV. In Section , we describe the basic concepts and fuzzification process of normalized delay, stability, and residual energy. In Section , we compare the performance of the fuzzy logic algorithm and the fuzzy-logic-assisted AODV algorithm with the traditional AODV and present the simulation results.

## 2. System Model and AODV Protocol

### 2.1. System Model

**Figure 1:**
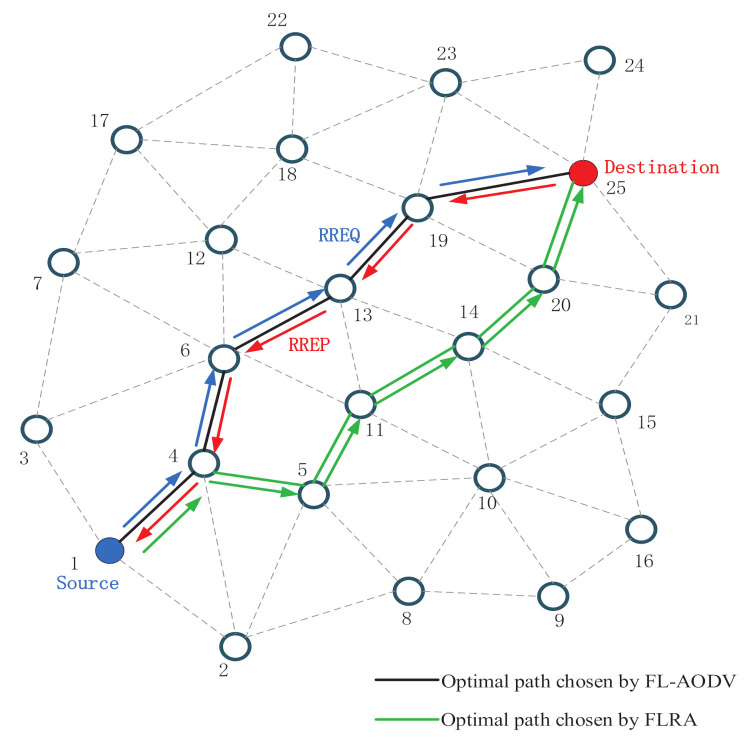
System model of establishing and evaluating the fuzzy-logic-assisted AODV routing protocol.

We consider establishing and evaluating the fuzzy-logic-assisted AODV routing protocol in the MANET environment, as shown in Figure 1. We assume that each mobile node is equipped with GPS and the same beam array antenna in order to know its position and speed information in real time. In the route discovery process, it is supposed that each antenna has its own node ID to release its position coordinates, which allows the receiving node to calculate the direction of the beam array antenna of the sending node, thereby avoiding flooding broadcast. When communication needs to be established, each mobile node can directionally receive the routing table of the neighboring node through the beam array antenna to estimate the information of the neighboring node. In addition, we assume that the switching time of the beam array antenna is negligible in this paper.

### 2.2. AODV Protocol

The AODV protocol is the most commonly used on-demand routing protocol [7]. The protocol performs the routing discovery process only when data need to be transmitted, which can relatively reduce the overhead of control messages [19], and can adapt to rapid changes in the topology [20]. The key technologies are the “request–response” mechanism and the sequence number mechanism, which can avoid routing loops. If the source node needs to transmit a message with a node in the network, first determine whether there is an available link to the destination node. If there is, use the available link for communication; otherwise, the node performs the path finding process. The source node starts to send routing requests to all neighboring nodes to find a path through RREQ. After receiving the RREQ, the intermediate node creates a reverse path to the source node. Then, continue to send the RREQ message. When the message reaches the destination node or the node containing the destination node information, the reverse route is created successfully. At the same time, the destination node generates a RREP message and transmits this message through the reverse path. After the source node receives the RREP message for the first time, the forward route is created, and the source node communicates with the destination node through this route.

However, in the process of path discovery, flooding and sending RREQ messages to surrounding nodes will cause serious control message overhead. In addition, the creation of a reverse route will cause a network storm and cause serious packet loss. In route selection process, the destination node only receives the first arrived RREQ message and establishes a forward route by responding to the source node with RREP packets. The process of establishing the route does not take the energy and stability of the nodes into consideration, which may result in the interruption of the link and in data loss and affects the routing reliability. In the following section, fuzzy logic is used to compromise multiple parameters to select the optimal relay node to establish the optimal route and enhance routing reliability.

## 3. Proposed Fuzzy-Logic-Assisted AODV Routing Protocol

Fuzzy logic has a relatively low computational overhead and performs well in dealing with nonlinear, time-varying, and unclearly defined processes. In this section, we describe the modeling of the fuzzy control system and the proposed routing algorithm based on fuzzy logic assistance.

### 3.1. Fuzzification

We focus on improving the route reliability based on the shortest path criterion of the traditional AODV protocol. In the route discovery process, we propose to select the most reliable node determined by the maximum output value of fuzzy logic system considering delay, stability, and remaining energy concurrently as the relay node. In the route selection process, the most reliable route path determined by the maximum cumulative sum of fuzzy logic output values of path is reserved for data transmission. In terms of fuzzification, since the triangular membership function has more intuitive and lower computational overhead [21], the input and output metrics in this paper choose the triangular membership function to calculate the fuzzy set. In addition, considering that the node of the normalized residual energy less than 10% easily cause link interruption and the energy consumption of the routing data transmission is not affected when it is higher than 90%, thus the suitable triangular and trapezoidal membership functions [22] are chosen to establish the normalized residual energy.

**Figure 2:**
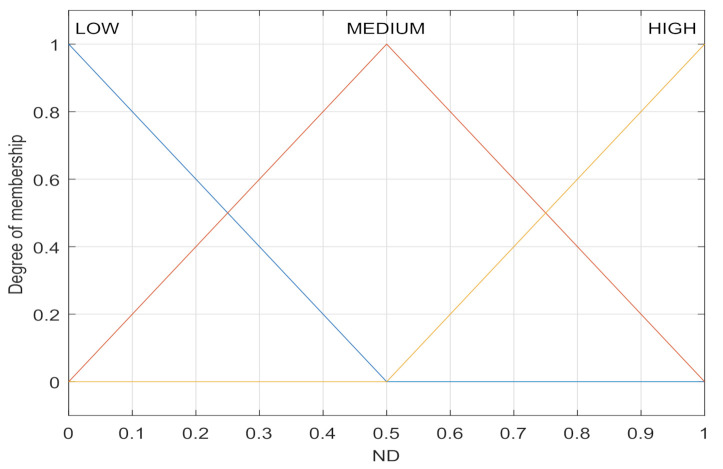
Membership function of ND.

The normalized delay (ND) of nodes is expressed as the average link delay with neighboring nodes, which can be given by [16]

(1) $$ND\left(x\right)=\frac{\sum_{i=1}^{n}NDx(y_{i}\in N_{x})}{n},$$

where NDx(y) is normalized link delay, NDx(y)=d(x,y)/R if d(x,y)<R, and NDx(y)=1; otherwise, $N_{x}$ denotes the neighbor nodes of the node *x*, *n* represents the number of neighbor nodes, d(x,y) is the distance between node *x* and node *y*, and *R* represents the longest distance that two nodes can communicate in this system. A triangular function is used to establish the membership function, as shown in Figure 2. The fuzzification process of ND is that the normalized delay value calculated according to Formula (1) is used as the abscissa, and the ordinate of the intersection point is the fuzzification language. For example, when the ND input is 0.4, the fuzzification language is {LOW:0.2, MEDIUM:0.8, HIGH:0}.

**Figure 3:**
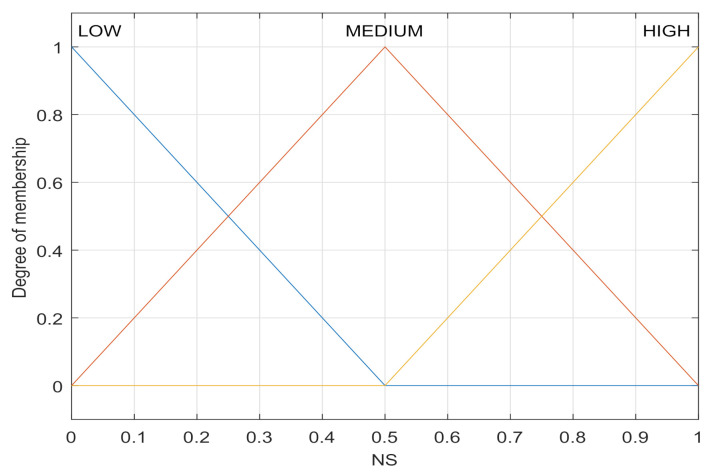
Membership function of NS.

The normalized stability (NS) of nodes [16] is given by

(2) $$NS\left(x\right)=1-\frac{\left|v\left(x\right)\right|-\min\left(y\in N\{|v(y)|\}\right)}{\max\left(y\in N\{|v(y)|\}\right)-\min\left(y\in N\{|v(y)|\}\right)},$$

where v(x) is the speed of the node *x* and *N* refers to the set of all nodes—the lower the speed of the node *x*, the higher the stability. A triangle function is used to establish the membership function of stability, as shown in Figure 3. The fuzzification process of NS, the normalized stability value calculated according to Formula (2), is used as the abscissa, and the ordinate of the intersection point is the fuzzification language. For example, when the NS input is 0.7, the fuzzification language is {LOW:0, MEDIUM:0.6, HIGH:0.4}.

**Figure 4:**
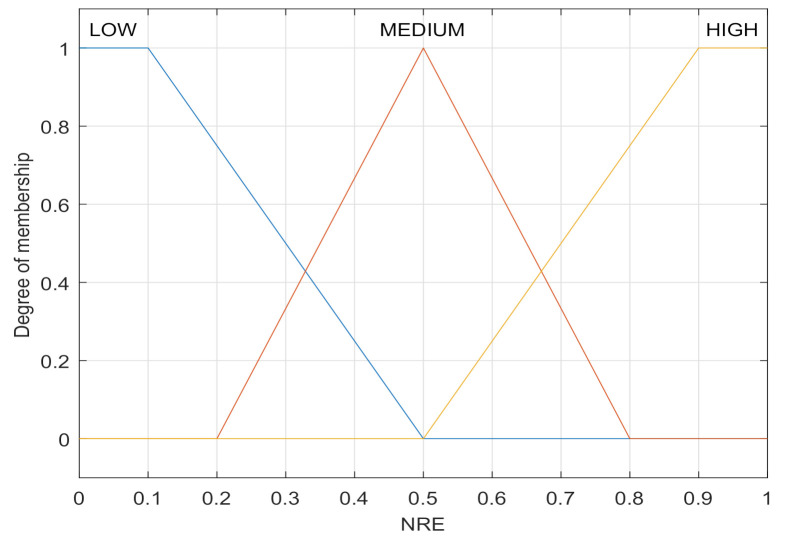
Membership function of NRE.

The normalized residual energy (NRE) is given by

(3) $$NRE\left(x\right)=\frac{E_{x}-\min\left(E_{y}\in N\left\{E\right\}\right)}{\max\left(E_{y}\in N\left\{E\right\}\right)-\min\left(E_{y}\in N\left\{E\right\}\right)},$$

where $E_{x}=E_{0}-v_{x}*t$ refers to the remaining energy of the node *x*, $E_{0}$ and $v_{x}$ are the initial energy and the energy consumption rate of the node *x*, respectively. A higher NRE means a higher possibility of being used as a relay node. Trapezoidal and triangular functions are used to establish the membership function of NRE, as shown in Figure 4. Select the fuzzy language when the remaining energy is less than 10 percent as {LOW:1, MEDIUM:0, HIGH:0}, and the fuzzy language with more than 90 percent as {LOW:0, MEDIUM:0, HIGH:1}. The fuzzification process of NRE is that the normalized residual energy value calculated according to Formula (3) is used as the abscissa, and the ordinate of the intersection point is the fuzzification language. For example, when the NRE input is 0.7, its fuzzification language is {LOW:0, MEDIUM:0.33, HIGH:0.5}.

### 3.2. Fuzzy Rules

**Figure 5:**
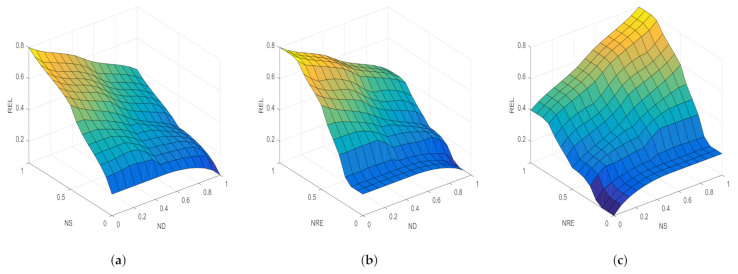
Input and output surface graph of fuzzy inference. (**a**) Inference surface between ND and NS and REL; (**b**) inference surface between ND and NRE and REL; (**c**) inference surface between NS and NRE and REL.

Fuzzy logic systems can use human experience and preferences to solve any complex problems. Fuzzy rules are a set of independent rules based on experience and simulation experiments to achieve certain effects  [15]. This paper chooses the “if–then” reasoning relationship. To realize high reliability, based on communication experience, the fuzzy rules set in this paper are shown in Table 1. It can be seen that the value of REL (reliability) increases with the decrease of ND and the increase of NS and NRE. Nodes with high REL value are most likely chosen as relay nodes. For example, according to QoS requirements and inference system, if the input delay is smaller, i.e., ND less than 0.1, the stability is higher, i.e., NS greater than 0.9, and the residual energy is more, i.e., NRE more than 0.9, the output reliability is higher, as shown in the yellow area of input and output inference surface graph in Figure 5. It is effective to avoid choosing nodes with low REL value as a relay node during the route discovery process.

### 3.3. Defuzzification

Defuzzification is to convert the fuzzy output language obtained by the fuzzy processing into an accurate output language. This paper uses the center of gravity (COG) [23] method to achieve defuzzification. The main theory of COG is to output the center of gravity of the membership function curve and the enclosed area of the abscissa as the final output value of the fuzzy control as

(4) $$v_{0}=\frac{\int_{v}v\mu_{v}\left(v\right)dv}{\int_{v}\mu_{v}\left(v\right)dv}.$$

**Figure 6:**
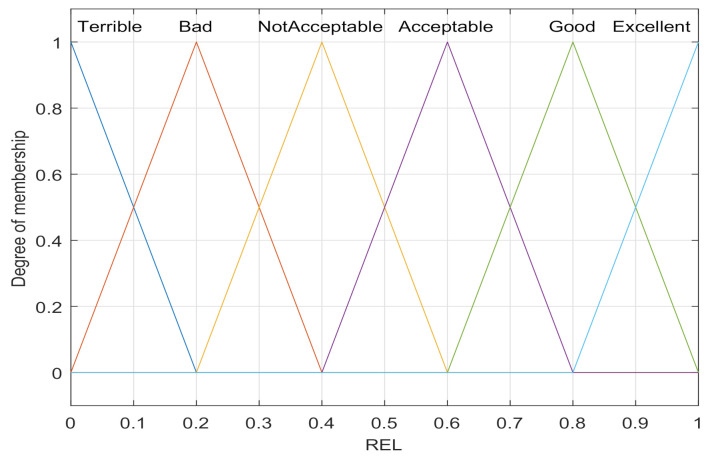
Membership function of REL.

The COG method has smoother output inference control. Even in response to a small change in the input signal, the output will change. In the paper, we select the output reliability level REL to comprehensively evaluate the three parameters of the node, and set the six fuzzy languages Excellent, Good, Acceptable, Not acceptable, Bad, and Terrible as the membership function of REL output, as shown in Figure 6. For example, when the fuzzy language calculated and output according to the input fuzzy processing part is {Not Acceptable:0.5, Acceptable:0.5}, the REL value of the output result of defuzzification according to the COG is 0.5.

### 3.4. Fuzzy System Realization Flow Chart

**Figure 7:**
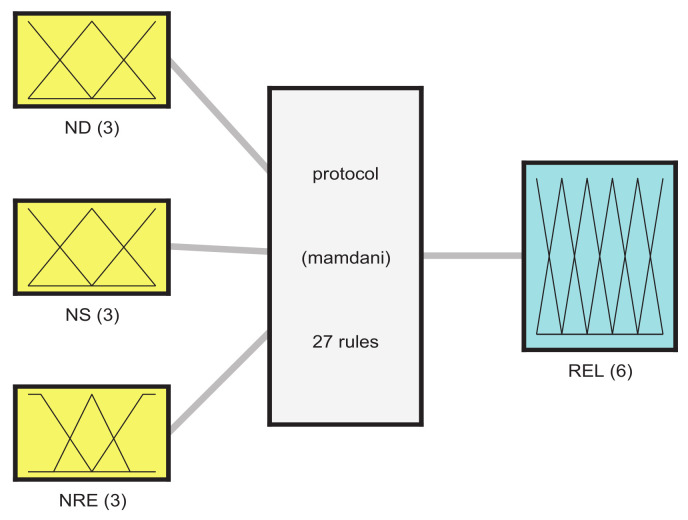
Flow chart of fuzzy system.

The fuzzy inference system established in this paper takes the normalized delay, stability, and residual energy value calculated by the node as input, and outputs the reliability level of the node after fuzzy processing. The higher the reliability, the more likely the node is chosen as a relay node. Through the fuzzy system shown in Figure 7, the relay node can be determined in real time. The choice of route can be determined by the highest average output reliability level of the route. Finally, a routing path with low latency, high link connectivity, and long network lifetime is obtained.

### 3.5. FL-AODV Protocol

The proposed FL-AODV routing algorithm selects the appropriate node by integrating multiple parameters, taking into account the reliability of the relay node, which solves the limitation of considering a single factor in the routing selection process. The proposed protocol is an extension of the AODV protocol and does not use additional control packets to collect network status information. Through the HELLO data packet, the information of all nodes can be collected, including the position, speed, and remaining energy of the node. Each node calculates the normalized delay, stability, and residual energy value by receiving the neighbor node information provided by the HELLO message of the neighbor node, and processes it through the fuzzy logic system, and calculates the reliability value of each node in real time. The FL-AODV protocol uses the reliability value provided by each node to find a route from the source node to the destination node.

**Figure 8:**
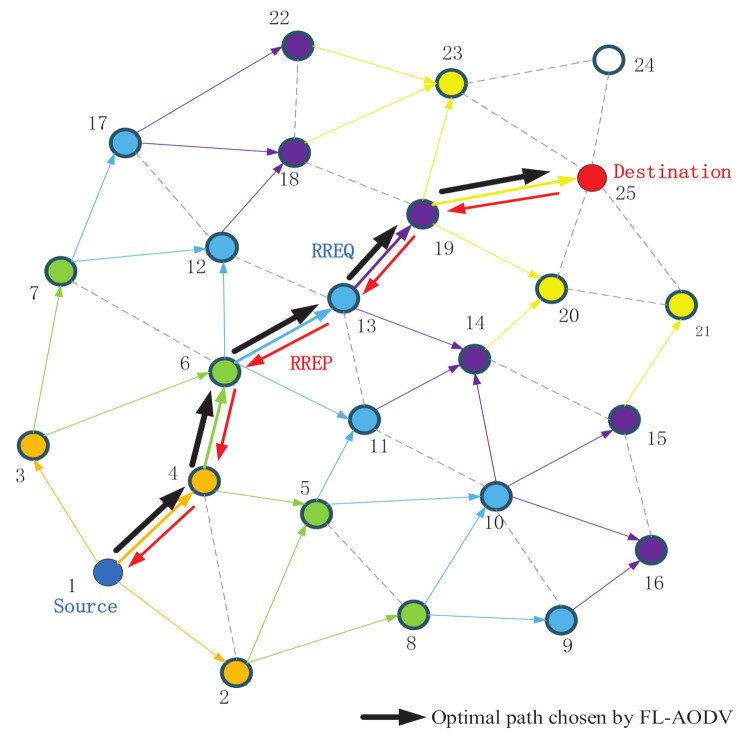
The route discovery process of FL-AODV.

In the route discovery process, the intermediate node selects the node with the highest REL value as the relay node, which can make the average reliability value of the selected path better than the path that only sets the RREQ received for the first time as the relay node and achieve better overall performance. In the route selection process, the destination node selects the node with the largest reliability value as the relay node, so the finally selected path has the largest average reliability value. In addition, the intermediate node will not forward the RREQ to the previous hop node that has forwarded the RREQ, different from the traditional flooding, which can reduce the flooding overhead accordingly. The specific routing process of the protocol is described in Figure 8.

First, source node 1 initializes REL value of the RREQ packet to 0 and updates its REL value to RREQ, and broadcasts RREQ to neighboring nodes, as shown by the orange nodes. Then, the neighboring nodes accumulate the REL value to update RREQ , and establish reverse routes.

Then, the relay nodes 2, 3, and 4 broadcast RREQ to neighbor nodes other than the source node, and the intermediate node selects the previous hop node that forwards the RREQ with the largest REL value among the received RREQs as the next hop of the reverse route. For example, node 6 will select a 4-to-1 path with a larger REL value, accumulate its REL value to RREQ, and establish a reverse route, as shown by the green path.

Similarly, the intermediate node continues to forward the RREQ to the neighbor nodes. Different from the AODV protocol, it will not send RREQ to the previous hop node that has forwarded the packet and the node with the same hop count, which reduces the huge overhead caused by complete flooding. In addition, the intermediate node will select the largest REL value of the fuzzy logic output as the relay node in the last hop node that forwards the RREQ to the node, update RREQ, and establish a reverse route, as shown by blue and purple path.

Last, the destination node 25 selects the route with the largest REL value as the optimal route, and according to the reverse route to unicast RREP packets to the source node, as shown by the red arrow.

In short, improved AODV algorithm based on the fuzzy logic is described in Algorithm (26).
**Algorithm 1
** Framework of relay nodes and routing selection based on fuzzy logic**Input:** Distance, speed and energy of nodes;**Output:** Route with high average reliability;1:Calculate the normalized delay (ND), stability (NS), and remaining energy (NRE) based on the distance, speed, and energy of the node;2:Input ND, NS, and NRE to the fuzzy system;3:Output value REL value according to the fuzzy rules established by the fuzzy system;4:Select the RREQ forwarding node with the largest REL value as the relay node;5:Superimpose REL value and forward;6:Repeat 4 and 5 until the receiving node is the destination node;7:return optimal route;


## 4. Simulation and Results

In order to verify the effectiveness of the proposed FL-AODV algorithm, we compare the FL-AODV algorithm with the traditional AODV and fuzzy logic routing algorithm (FLRA). As an algorithm with common feature of fuzzy-logic-based routing algorithm in exiting work, FLRA algorithm, only uses fuzzy logic to find the route and select the neighbor node with the highest reliability value of fuzzy logic output as the relay node. Until the destination node is located in the neighbor node, the destination node with higher priority is selected to construct the optimal route, as shown by green path in Figure 1. Different from the traditional AODV algorithm which takes the path to the destination node first as the routing rule, i.e., the shortest path criterion and the FLRA algorithm that only relies on reliability to select the route, the FL-AODV algorithm selects the path with lower delay, higher stability, and more residual energy as the optimal path on the basis of AODV and fuzzy logic.

The simulation environment parameters are set to a square area of 1000 m × 1000 m, in which nodes are randomly distributed. The remaining energy of the nodes varies with time and energy consumption rate gradually decrease and the energy consumption rate of each node is evenly distributed. In this paper, we analyze and compare the performance of the appropriate path selected by the three algorithms in exactly the same scene. The performance indicators choose the average path reliability, end-to-end delay, hop count, link connectivity, and route life as the analysis objects. Detailed simulation parameter settings are given in Table 2.

**Figure 9:**
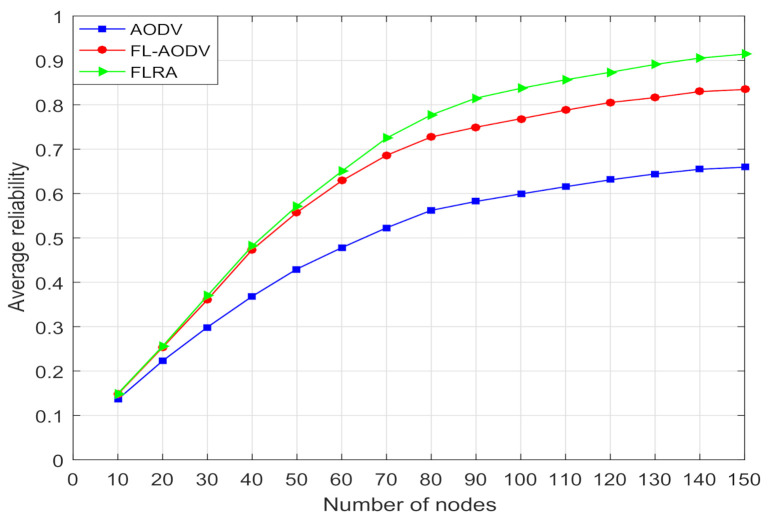
Comparison of average reliability of paths.

In Figure 9, the average reliability calculated mainly by stability and residual energy of the three algorithms are compared. It can be seen that, when the number of nodes is less than 30, the average reliability of the three algorithms is too low owing to the high probability of link interruption, and increases slowly when the number of nodes is greater than 80, because the reliability is less affected by the link interruption in dense networks. As the number of nodes increases, due to the relay node selection of the highest reliability, the average routing reliability of the FLRA algorithm is better than the other two algorithms, while AODV algorithm performs worse without considering the reliability. Compared with the traditional AODV routing algorithm, the proposed FL-AODV algorithm, which considers both routing reliability and the shortest path concurrently, is improved by about 18% when the number of nodes is greater than 70 in terms of average path reliability .

**Figure 10:**
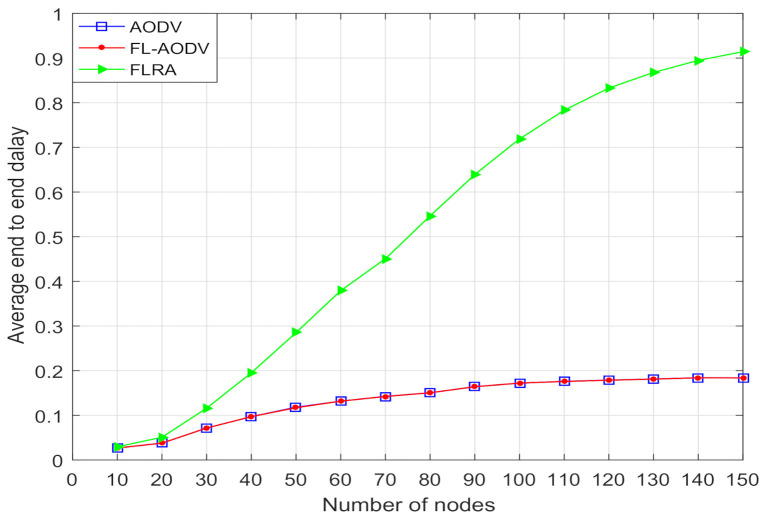
Comparison of average end-to-end latency.

**Figure 11:**
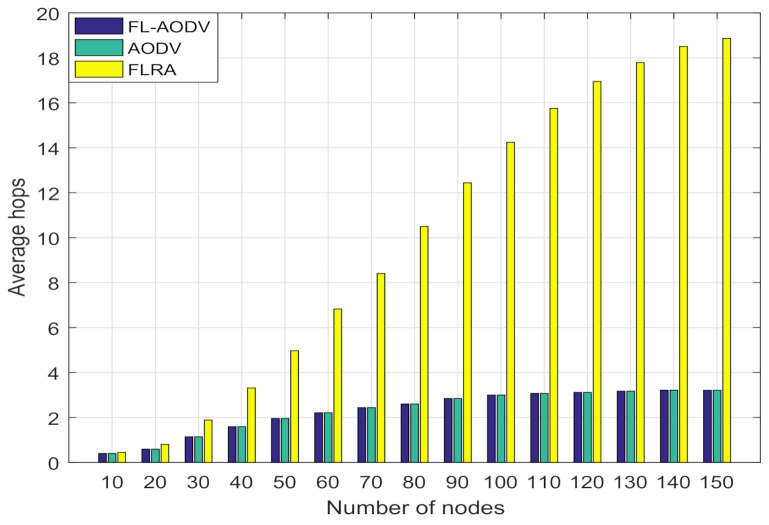
Comparison of average path hops.

In Figure 10 and Figure 11, we compare the average end-to-end latency and path hops of the three algorithms. It can be seen that, with increases in node number, the average end-to-end latency and path hops of the FL-AODV and AODV gradually increase and approach a stable value when the number of nodes is more than 90, while the FLRA increases rapidly. This phenomenon occurs due to more relay nodes are selected in the path discovery process of the FLRA algorithm as the number of nodes gradually increases, the average number of hops increases sharply, resulting in a rapid increase in average end-to-end delay. However, since the shortest path characteristics of AODV, the FL-AODV algorithm similar to the AODV algorithm maintains lower latency and hops.

**Figure 12:**
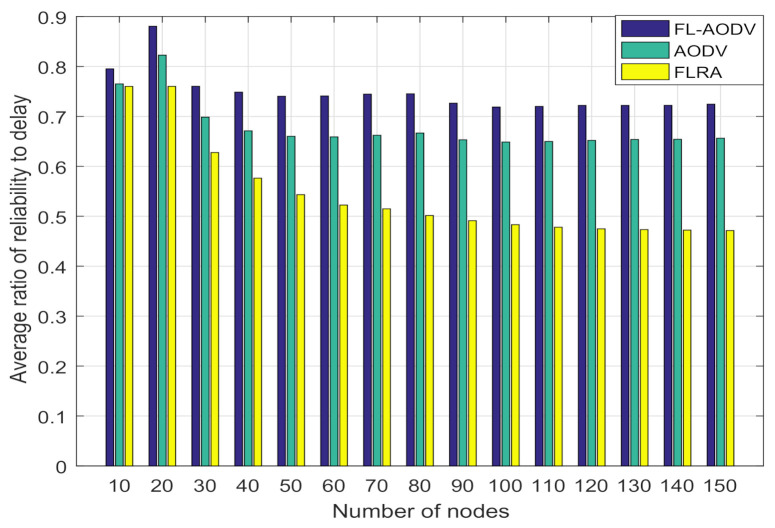
Comparison of average reliability to delay ratio.

In order to better compare the overall performance of these three algorithms on reliability and delay, we construct the ratio of reliability to delay, where it is proportional to the average reliability, and inversely proportional to the average end-to-end delay. As can be seen from the comparison of reliability to delay ratio in Figure 12, as the number of nodes is gradually increased, the comprehensive performance of FL-AODV performs better, while the FLRA performance is worse. This confirms that FL-AODV is better to achieve high reliability and low latency performance, although the reliability of FLRA is higher than the FL-AODV algorithm as shown in Figure 9. Especially, the FL-AODV algorithm is about 10% higher than the AODV algorithm and 25% higher than the FLRA algorithm when the number of nodes is greater than 100 in terms of ratio of reliability to delay. In short, compared traditional AODV and FLRA, the proposed algorithm FL-AODV has a better overall performance.

Moreover, in order to verify that the proposed algorithm has better link connectivity, longer route life, and scalability in terms of speed at the same time, in this paper, we compare the average link connectivity and route life of these three algorithms at different moving speeds of nodes.

**Figure 13:**
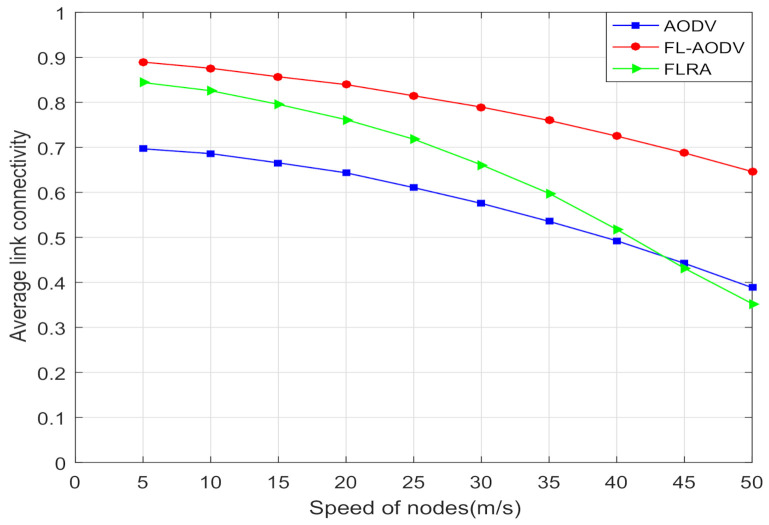
Comparison of average link connectivity at different speeds.

It can be seen from Figure 13 that the average link connectivity determined mainly by link interruption probability and routing reliability of these three algorithms decrease with the increase in maximum moving speed owing to the greater randomness of selection of relay nodes and the routing of source and destination nodes in a network with rapidly changing nodes. Compared with AODV and FLRA, the FL-AODV algorithm performs better. It is because that the reliability of AODV algorithm is worse than the other two algorithms optimized by fuzzy logic, which results in lower link connectivity, and the link interruption probability of FLRA algorithm is more prominent due to greater randomness of selection of nodes when the speed is greater than 25 m/s, which accelerates the decline of the link connectivity. However, the reliability and link interruption probability of FL-AODV change slower, therefore, with the increase of node speed, the FL-AODV algorithm achieves the better average link connectivity.

**Figure 14:**
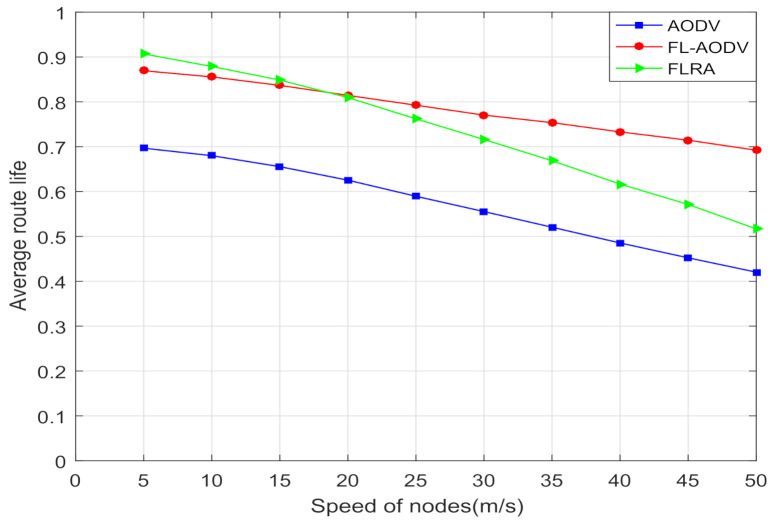
Comparison of average route life at different speeds.

In Figure 14, the average route life determined mainly by residual energy and routing reliability of the three algorithms are compared. It can be seen that, compared to AODV and FLRA, the route life of the FL-AODV algorithm performs better with the increase in maximum moving speed. It occurs because, as the speed increases, the nodes change rapidly, the routing reliability, and residual energy of the FLRA algorithm that only selects the best reliable node is greatly affected, thereby reducing the route life rapidly. In addition, since the residual energy and reliability of the node is not considered, the routing life of AODV is shorter. However, the route reliability of FL-AODV algorithm based on AODV is less affected owing to the feature of the shortest path. Therefore, with the increase of node speed, the FL-AODV algorithm achieves the longer route life.

In summary, the overall performance of the FL-AODV algorithm is better since it has higher reliability including better link connectivity and longer route life under the condition of low delay and low hop count. Besides low complexity, fuzzy logic comprehensively considers multiple indicators for routing selection, and thus can better meet the increasing demand for higher communication service quality.

## 5. Conclusions

In this paper, we proposed a highly reliable fuzzy-logic-assisted AODV routing algorithm. The proposed algorithm comprehensively considered delay, stability, and residual energy as the routing criteria, and realized the selection of a low-latency, high-link connectivity, and long-life routing through a fuzzy system. Simulation results verified that, compared with traditional AODV and FLRA algorithm, the proposed algorithm has a higher reliability without increasing delay, i.e., better link connectivity and longer route life and is more suitable for the scenarios where users are moving at high speed. Especially, the FL-AODV algorithm is about 10% higher than the AODV algorithm and 25% higher than the FLRA algorithm when the number of nodes is greater than 100 in terms of ratio of reliability to delay, which confirms that FL-AODV algorithm can better achieve high reliability and low latency performance.

## Figures and Tables

**Table 1:** Fuzzy rules.

Rules	ND	NS	NRE	REL
1	LOW	HIGH	HIGH	Excellent
2	LOW	HIGH	MEDIUM	Good
3	LOW	HIGH	LOW	Not Acceptable
4	LOW	MEDIUM	HIGH	Good
5	LOW	MEDIUM	MEDIUM	Acceptable
6	LOW	MEDIUM	LOW	Bad
7	LOW	LOW	HIGH	Not Acceptable
8	LOW	LOW	MEDIUM	Bad
9	LOW	LOW	LOW	Terrible
10	MEDIUM	HIGH	HIGH	Good
11	MEDIUM	HIGH	MEDIUM	Acceptable
12	MEDIUM	HIGH	LOW	Bad
13	MEDIUM	MEDIUM	HIGH	Acceptable
14	MEDIUM	MEDIUM	MEDIUM	Not Acceptable
15	MEDIUM	MEDIUM	LOW	Bad
16	MEDIUM	LOW	HIGH	Not Acceptable
17	MEDIUM	LOW	MEDIUM	Bad
18	MEDIUM	LOW	LOW	Terrible
19	HIGH	HIGH	HIGH	Acceptable
20	HIGH	HIGH	MEDIUM	Not Acceptable
21	HIGH	HIGH	LOW	Terrible
22	HIGH	MEDIUM	HIGH	Not Acceptable
23	HIGH	MEDIUM	MEDIUM	Bad
24	HIGH	MEDIUM	LOW	Terrible
25	HIGH	LOW	HIGH	Bad
26	HIGH	LOW	MEDIUM	Terrible
27	HIGH	LOW	LOW	Terrible

**Table 2:** Parameter setup.

Parameters	Value
Simulator	MATLAB
Number of nodes	10–150
Scope of broadcasting	200 m
Simulation Area	1000 m × 1000 m
Speed of nodes	0–10 m/s
Routing algorithm	FL-AODV, AODV, FLRA

## Data Availability

Not applicable.
